# Eculizumab as salvage therapy for recurrent monoclonal gammopathy-induced C3 glomerulopathy in a kidney allograft

**DOI:** 10.1186/s12882-018-0904-7

**Published:** 2018-05-03

**Authors:** Philipp Moog, Philipp J. Jost, Maike Büttner-Herold

**Affiliations:** 1 Department of Nephrology, Klinikum rechts der Isar, Technische Universität München, Ismaninger Str. 22, 81675 Munich, Germany; 2 III. Medizinische Klinik, Klinikum rechts der Isar, Technische Universität München, Munich, Germany; 3 Department of Nephropathology, Institute of Pathology, Universitätsklinikum Erlangen, Friedrich-Alexander-Universität Erlangen-Nürnberg, Erlangen, Germany

**Keywords:** Monoclonal gammopathy, Renal transplantation, Acquired complement disorders, Eculizumab

## Abstract

**Background:**

Monoclonal gammopathy causes several kinds of renal pathology. A rare and special form is monoclonal gammopathy-induced C3 glomerulopathy (MG-C3G). Like idiopathic C3G, MG-C3G frequently leads to end-stage renal disease. MG-C3G frequently recurs after renal transplantation, leading to graft failure in most of the patients. While there is some evidence for successful treatment of recurrent idiopathic C3 glomerulopathy with eculizumab after renal transplantation, nothing is known about its efficacy in the setting of recurrent MG-C3G.

**Case presentation:**

We report a patient with recurrent MG-C3G in a renal allograft that was successfully treated with eculizumab in addition to standard immunosuppression. He had early recurrence of MG-C3G 2 months after transplantation. His graft function successively declined despite high dose steroids and plasmapheresis. Only after therapy with three cycles of bortezomib and continuous therapy with eculizumab, his graft function stabilized. He was still in clinical remission after 28 months of follow-up without having experienced major infectious complications.

**Conclusions:**

Eculizumab may be a safe and effective treatment of recurrent MG-C3G. Because of the high and early recurrence risk, renal transplantation should be reviewed carefully for every individual patient. Subsequent hematopoietic stem cell transplantation may ameliorate long-term renal allograft survival. Eculizumab might serve as a bridging therapy until stem cell transplantation.

## Background

C3 Glomerulopathies (C3G) result from abnormal regulation of the alternative complement pathway (AP) with uncontrolled C3b amplification and subsequent damage by complement induced glomerular inflammation [1]. Both hereditary and acquired complement defects that induce this rare form of glomerulonephritis have been identified. A special form of acquired C3G has been described in patients with monoclonal gammopathy [2–5]. Several mechanisms may lead to uncontrolled AP activation in monoclonal gammopathy. Like in a non-myeloma setting of C3G, C3 nephritic factor (C3NeF) and antibodies against factor H can be detected [3]. Moreover, circulating monoclonal lambda light chains can act as a mini antibody against factor H, leading to uncontrolled perpetuation of complement activation [6].

Recurrence of C3G is a major problem after renal transplantation. It occurs in more than 50% of patients within 5 years and is responsible for up to 70% of graft failure [7, 8]. According to the available literature, disease recurrence seems to have an even higher impact on graft function and graft survival in patients with MG-C3G. In a cohort of patients with C3G that received a kidney transplant, three patients had underlying monoclonal gammopathy. All three patients had recurrent disease after a median of 3.6 months compared to median time to recurrence of 43.3 months in patients without monoclonal gammopathy [9]. In a recent report, all four patients that received a kidney transplant had disease recurrence after 3 to 12 months, leading to graft failure in one patient [3].

Some evidence from case reports suggests that eculizumab, a monoclonal C5-antibody, may be an effective treatment for C3G [10–17]. It has also been applied successfully in three published cases of recurrent C3G after renal transplantation [18–20]. However, experience with eculizumab in the setting of recurrent MG-C3G after renal transplantation is lacking.

We herein describe a patient with recurrence of MG-C3G in a kidney allograft that was successfully treated with eculizumab, leading to sustained recovery after dialysis-dependent allograft failure.

## Case presentation

A 59-year-old male patient was diagnosed in 2003 with a smoldering multiple myeloma (IgG lambda) with bone marrow infiltration of 10 to 20% (negative CRAB criteria at that time). In 2004, he developed nephrotic syndrome and a histological diagnosis of focal segmental glomerulosclerosis was made. Immunofluorescence showed C3c deposition but dense deposits were absent on electron microscopy. Six months later, the patient developed progressive renal failure with nephritic syndrome. A subsequent kidney biopsy revealed crescentic membranoproliferative glomerulonephritis with dense deposits (dense deposit disease; DDD). Immunohistochemistry was negative for IgG, IgA, IgM and C1q. Despite treatment with cyclophosphamide, his kidney function rapidly decreased to end-stage renal disease (ESRD) in 2004. In the light of a stable remission of his multiple myeloma for 10 years (stage I Salmon/Durie; stage III ISS in 2014; positive CRAB-criteria: renal failure, anemia), a kidney transplantation was planned.

**Fig. 1:**
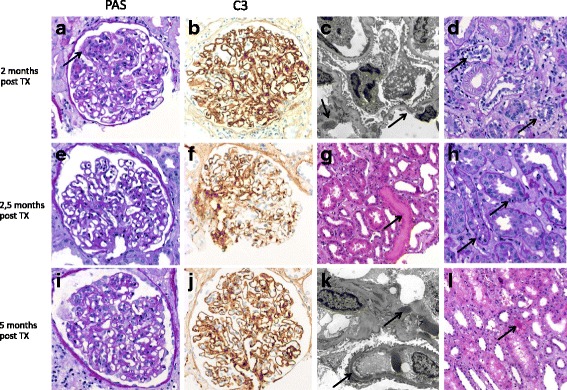
**a**-**d** Kidney biopsy 2 months post TX with mild intracapillary proliferation (arrow, **a**) and intense mesangiocapillary C3-deposit (**b**). In EM mesangial and intramembranous osmiophilic deposits are seen (arrows, **c**). Additionally, moderate tubulitis was present indicative of acute cellular rejection Banff IA (**d**). **e**-**h** Kidney biopsy 2,5 months post TX with persistent C3-dominant GN (**e**, **f**) signs of hematuria with erythrocyte cylinders (arrow, **g**) and only mild persistent tubulitis diagnostic of borderline cellular rejection (arrows, **h**). **i**-**l** Kidney biopsy 5 months after TX showing intracapillary proliferative GN (**a**) with persistently intense C3-deposits (**j**) and osmiophilic deposits in the lamina densa of the glomerular basement membrane and the mesangia (**k**). Additionally signs of hematuria (arrow, **l**). All LM pictures were taken with an AxioCam MRc and an Imager. A1 Axio microscope (Zeiss, Germany). **a**, **b**, **d**, **e**, **f**, **h**, **i**, **j** at 400× original magnification (o.m.), **g**, **l** 200× o.m. EM pictures (**c**, **k**) taken at a 5000× o.m. with a Leo912 electron microscope (Zeiss, Germany)

**Fig. 2:**
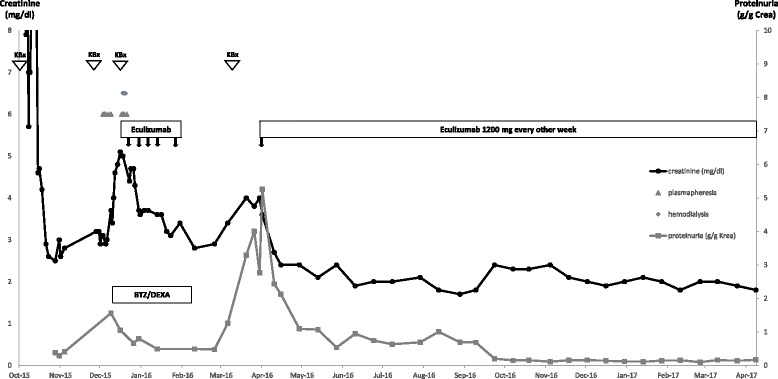
Course of creatinine and proteinuria after the first allograft rejection. Abbreviations: KBx: Kidney biopsy; BTZ: bortezomib; DEXA: dexamethasone

After deceased donor kidney transplantation in October 2015, a kidney biopsy was performed at day seven because of delayed graft function. Immunosuppressive medication consisted of cyclosporine, mycophenolate mofetile and oral glucocorticoids without prior induction therapy. The biopsy revealed acute tubular necrosis, acute cellular rejection and an intracapillary proliferative glomerulonephritis (Banff IA; Fig. 1 a-d). The biopsy result before receiving the additional immunohistochemical staining was compatible with mixed cellular and humoral rejection. The patient received four sessions of plasmapheresis and three doses of antithymocyte globuline (75 mg each) in combination with glucocorticoid pulse therapy. Cyclosporine was switched to tacrolimus. Additional immunohistochemical stainings revealed C3c deposition and on electron microscopy mesangial and dense intramembranous osmiophilic deposits were present, indicating an early recurrence of DDD in the allograft (Fig. 1 a-d). Graft function improved to a stable creatinine of 2.6 mg/dl over several weeks (Fig. 2).

In December 2015, creatinine increased to 3.2 mg/dl. Serum C3 was low (55 mg/dl) and the patient had active urine sediment. Tacrolimus trough levels were stable. A successive kidney biopsy confirmed C3G and minimal residual tubulitis diagnostic of borderline cellular rejection (Fig. 1 e-h). CRAB criteria were positive for renal insufficiency and anemia. The concentration of free lambda light chain was 10.2 mg/l. Another course of high-dose corticosteroids was combined with five sessions of plasmapheresis and subsequent induction therapy with bortezomib and dexamethasone. Myeloma bone marrow infiltration was 20% at that time. After short-term stabilization (creatinine 3.2 mg/dl over 14 days), graft function rapidly deteriorated over several days (Fig. 2). Despite four sessions of plasmapheresis, allograft function further declined and hemodialysis treatment was startet on 30th Dec 2015. Because of refractory MG-C3G, treatment was switched to eculizumab. The first infusion of 900 mg was given on 4th Jan 2016 followed by three infusions of 900 mg eculizumab weekly and one additional dose of 1200 mg 2 weeks thereafter. Graft function improved promptly and tapering of oral steroids was possible. Serum C3 increased from 55 mg/dl before treatment to 84 mg/dl after the induction course of eculizumab. Bortezomib had to be stopped after three cycles because of polyneuropathy.

In March 2016, creatinine increased and the patient developed nephrotic-range proteinuria (Fig. 2). A graft biopsy showed active DDD without any sign of myeloma infiltration (Fig. 1). The myeloma bone marrow infiltration at this time was 6% and free lambda light chain concentration was 10.4 mg/l. After restarting eculizumab 1200 mg every other week, creatinine and proteinuria improved quickly, indicating a rapid clinical response to complement inhibition. At the last follow-up in March 2018, the patient was well and had a stable allograft function (creatinine of 1.9 mg/dl) without proteinuria (230 mg/g creatinine). Serum C3 was decreased with 57 mg/dl, indicating persisting subclinical immunological activity of MG-C3G. Free lambda light chain concentration at last follow-up was 8.9 mg/l. No progression of myeloma occurred during a 28-months-follow-up after kidney transplantation and besides bortezomib-induced polyneuropathy, no adverse treatment effects were observed.

## Discussion

To our knowledge, we report for the first time that eculizumab, in combination with bortezomib and standard immunosuppression, may lead to sustained stabilization of allograft function in recurrent MG-C3G. In keeping with previous reports our patient had early recurrence of MG-C3G 2 months after renal transplantation. Standard immunosuppression in combination with plasmapheresis was not sufficient to reverse allograft dysfunction. Only after treatment with bortezomib and eculizumab, a marked and prolonged improvement of allograft function was achieved. However, the early recurrence after a break in eculizumab therapy and the persistent reduction in serum C3 indicate ongoing complement activation by the monoclonal protein. Hence, continuous therapy may be needed for a sustained response in our and similar cases. Interestingly, bortezomib was not required in addition to eculizumab to achieve a clinical response after the second relapse. These observations may raise the question whether renal allograft survival would be more favorable with combined or subsequent hematopoetic stem cell transplantation and whether bortezomib plus eculizumab might serve as a bridging treatment until hematopoetic stem cell transplantation. With regard to the high risk of recurrent MG-C3G and associated graft failure, the indication for renal transplantation should be thoroughly reviewed in every individual patient.

## Conclusions

Eculizumab may be an effective and safe add-on therapy in cases of recurrent MG-C3G after kidney transplantation.

## Abbreviations

C3G: C3 Glomerulopathies
C3NeF: C3 nephritic factor
CRAB: C – hypercalcemia; R – renal failure; A – anemia; B – bone lesion
DDD: Dense deposit disease
ESRD: End stage renal disease
ISS: International staging system
MG-C3G: Monoclonal gammopathy induced C3 glomerulopathy

## References

[1] Barbour TD, Ruseva MM, Pickering MC (2016). Update on C3 glomerulopathy. Nephrol Dial Transplant.

[2] Bridoux F, Desport E, Frémeaux-Bacchi V, Chong CF, Gombert JM, Lacombe C, Quellard N, Touchard G (2011). Glomerulonephritis with isolated C3 deposits and monoclonal gammopathy: a fortuitous association?. Clin J Am Soc Nephrol.

[3] Chauvet S, Frémeaux-Bacchi V, Petitprez F, Karras A, Daniel L, Burtey S, Choukroun G, Delmas Y, Guerrot D, François A (2017). Treatment of B-cell disorder improves renal outcome of patients with monoclonal gammopathy-associated C3 glomerulopathy. Blood.

[4] Sethi S, Sukov WR, Zhang Y, Fervenza FC, Lager DJ, Miller DV, Cornell LD, Krishnan SG, Smith RJ (2010). Dense deposit disease associated with monoclonal gammopathy of undetermined significance. Am J Kidney Dis.

[5] Sethi S, Rajkumar SV (2013). Monoclonal gammopathy-associated proliferative glomerulonephritis. Mayo Clin Proc.

[6] Jokiranta TS, Solomon A, Pangburn MK, Zipfel PF, Meri S (1999). Nephritogenic lambda light chain dimer: a unique human miniautoantibody against complement factor H. J Immunol.

[7] Lu DF, Moon M, Lanning LD, McCarthy AM, Smith RJ (2012). Clinical features and outcomes of 98 children and adults with dense deposit disease. Pediatr Nephrol.

[8] Braun MC, Stablein DM, Hamiwka LA, Bell L, Bartosh SM, Strife CF (2005). Recurrence of membranoproliferative glomerulonephritis type II in renal allografts: the North American pediatric renal transplant cooperative study experience. J Am Soc Nephrol.

[9] Zand L, Lorenz EC, Cosio FG, Fervenza FC, Nasr SH, Gandhi MJ, Smith RJ, Sethi S (2014). Clinical findings, pathology, and outcomes of C3GN after kidney transplantation. J Am Soc Nephrol.

[10] Bomback AS, Smith RJ, Barile GR, Zhang Y, Heher EC, Herlitz L, Stokes MB, Markowitz GS, D'Agati VD, Canetta PA (2012). Eculizumab for dense deposit disease and C3 glomerulonephritis. Clin J Am Soc Nephrol.

[11] Herlitz LC, Bomback AS, Markowitz GS, Stokes MB, Smith RN, Colvin RB, Appel GB, D'Agati VD (2012). Pathology after eculizumab in dense deposit disease and C3 GN. J Am Soc Nephrol.

[12] Daina E, Noris M, Remuzzi G (2012). Eculizumab in a patient with dense-deposit disease. N Engl J Med.

[13] Inman M, Prater G, Fatima H, Wallace E (2015). Eculizumab-induced reversal of dialysis-dependent kidney failure from C3 glomerulonephritis. Clin Kidney J.

[14] Le Quintrec M, Lionet A, Kandel C, Bourdon F, Gnemmi V, Colombat M, Goujon JM, Frémeaux-Bacchi V, Fakhouri F (2015). Eculizumab for treatment of rapidly progressive C3 glomerulopathy. Am J Kidney Dis.

[15] Ozkaya O, Nalcacioglu H, Tekcan D, Genc G, Meydan BC, Ozdemir BH, Baysal MK, Keceligil HT (2014). Eculizumab therapy in a patient with dense-deposit disease associated with partial lipodystropy. Pediatr Nephrol.

[16] Radhakrishnan S, Lunn A, Kirschfink M, Thorner P, Hebert D, Langlois V, Pluthero F, Licht C (2012). Eculizumab and refractory membranoproliferative glomerulonephritis. N Engl J Med.

[17] Vivarelli M, Pasini A, Emma F (2012). Eculizumab for the treatment of dense-deposit disease. N Engl J Med.

[18] McCaughan JA, O'Rourke DM, Courtney AE (2012). Recurrent dense deposit disease after renal transplantation: an emerging role for complementary therapies. Am J Transplant.

[19] Sánchez-Moreno A, De la Cerda F, Cabrera R, Fijo J, López-Trascasa M, Bedoya R, Rodríguez de Córdoba S, Ybot-González P (2014). Eculizumab in dense-deposit disease after renal transplantation. Pediatr Nephrol.

[20] Gurkan S, Fyfe B, Weiss L, Xiao X, Zhang Y, Smith RJ (2013). Eculizumab and recurrent C3 glomerulonephritis. Pediatr Nephrol.

